# Mu-seq: Sequence-Based Mapping and Identification of Transposon Induced Mutations

**DOI:** 10.1371/journal.pone.0077172

**Published:** 2013-10-23

**Authors:** Donald R. McCarty, Sue Latshaw, Shan Wu, Masaharu Suzuki, Charles T. Hunter, Wayne T. Avigne, Karen E. Koch

**Affiliations:** Horticultural Sciences Department, Plant Molecular and Cellular Biology Program, Genetics Institute, University of Florida, Gainesville, Florida, United States of America; University of Michigan, United States of America

## Abstract

Mutations tagged by transposon insertions can be readily mapped and identified in organisms with sequenced genomes. Collections of such mutants allow a systematic analysis of gene function, and can be sequence-indexed to build invaluable resources. Here we present Mu-seq (Mutant-seq), a high-throughput NextGen sequencing method for harnessing high-copy transposons. We illustrate the efficacy of Mu-seq by applying it to the *Robertson’s Mutator* system in a large population of maize plants. A single Mu-seq library, for example, constructed from 576 different families (2304 plants), enabled 4, 723 novel, germinal, transposon insertions to be detected, identified, and mapped with single base-pair resolution. In addition to the specificity, efficiency, and reproducibility of Mu-seq, a key feature of this method is its adjustable scale that can accomodate simultaneous profiling of transposons in thousands of individuals. We also describe a Mu-seq bioinformatics framework tailored to high-throughput, genome-wide, and population-wide analysis of transposon insertions.

## Introduction

Insertional mutagenesis has provided a vital foundation for development of large-scale collections of sequence-indexed mutants for model organisms. Advances in building and mining these resources have had a major impact on functional genomics research across a range of species from *Drosophila* [1], [2] to plants. The latter include a nearly comprehensive collection of T-DNA insertion lines for Arabidopsis [3], [4], transposon-based resources for rice [5]–[7], and in maize, the *Robertson’s Mutator* transposon has been widely used for both forward and reverse genetics [8]–[14]. The Mu transposon is a highly effective mutagen due to its preferential targeting of gene sequences [9], [15] and its capacity to attain a high-copy number in individual genomes [16]. The UniformMu maize population was designed specifically for functional genomics applications, and was constructed by backcross introgression of MuDR, the autonomous element of the canonical Robertson’s Mutator system [17], into an inbred genetic background [11]. Key features of the UniformMu resource include a built-in marker system that enables genetic control Mu transposition activity, a uniform inbred genetic background that facilitates phenotype analysis, and known pedigrees of all individuals in the population [11]. Efforts to create a searchable, sequence-index of transposons in the resource [11]–[13] highlighted the need for an efficient, reproducible, sequence-based method that could concurrently handle genome-wide discovery and mapping of Mu transposons in thousands of plants.

Mu transposons share a highly-conserved, terminal inverted repeat (TIR) sequence that is present at both ends of the element and is thus well-suited for design of Mu-specific primers [11], [12]. However, detection of genomic sequences flanking new insertions of interest can be challenging amid the complex background of pre-existing, Mu-related transposon sequences endogenous to the maize genome [18]. This background includes canonical Mu elements that are potentially activated by MuDR, as well as hundreds of sequences from divergent families of Mu-related transposons that may or may not remain active. Collectively, these divergent, Mu-related transposon sequences outnumber the active elements of greatest interest for functional genomics. The background of abundant endogenous Mu’s is especially challenging for applications using pooled samples, because new insertions will be still rarer due to their presence in a single individual. These insertions must be reliably detectable among the many-fold greater numbers of endogenous transposon insertions. Here we describe Mu-seq (Mutant-seq), a sequence-based method for high-throughput identification and mapping of transposon insertion sites, and demonstrate its efficacy using the *Robertson’s Mutator* system in the maize genome. We show that the specificity, efficiency, and single-base precision of the Mu-seq method are reproducible and ideally suited for genome-wide mapping of new transposon insertions. Mu-seq thus enables thousands of sequence-indexed mutants to be identified, mapped, and assigned to individuals in a large population. Mu-seq sequences derived from the background of pre-existing transposon insertions in the genome are subtracted *in silico* using a dedicated bioinformatics infrastructure. The Mu-seq approach is readily adaptable to diverse transposon systems and species.

## Results

### Anchoring Mu-seq Sequences to Transposons

**Figure 1:**
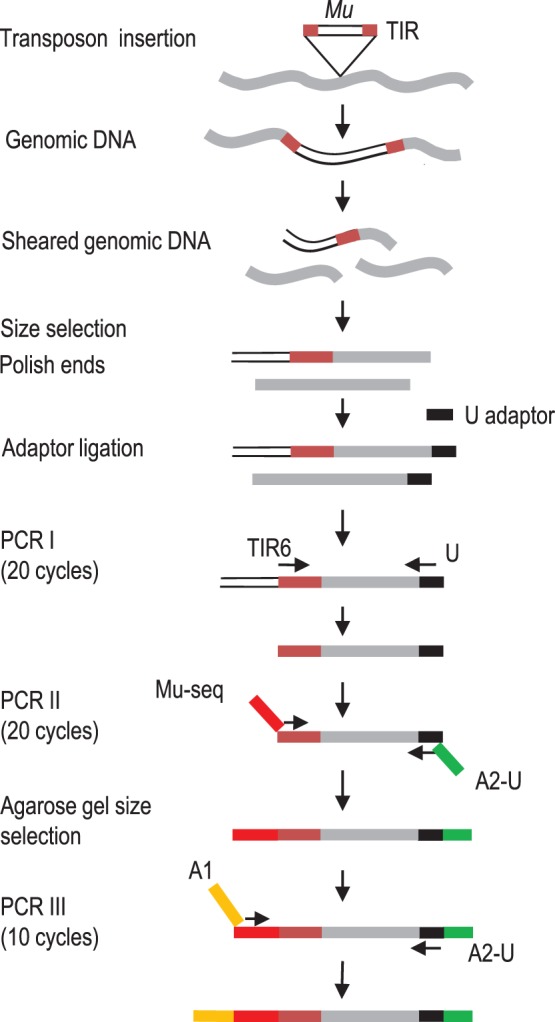
Protocol for construction of Mu-seq libraries. Genomic DNA from pools of 125 or more plants is sheared by sonication, size-enriched, made blunt-ended and ligated to an adaptor (U). The PCR I step uses a Mu-specific, TIR6 primer and a universal U adaptor primer to amplify genomic DNA fragments with TIRs at their 5′ ends. PCR II incorporates at the 5′-end, a Mu-seq primer that includes, from 5′ to 3′, part of the Illumina A1 sequencing adaptor, a 4-bp barcode, and a MuTIR region; and, at the 3′ end, an Ilumina, A2-U sequencing adaptor. PCR II products are size-selected by agarose gel electrophoresis. PCR III completes the nested synthesis of a complete A-1 Illumina sequencing adaptor. Colors highlight the TIR of a Mu transposon (brown), genomic DNA (grey), a universal adaptor U (black), the Mu-seq adaptor (red), an Illumina A2-U sequencing adaptor (green), and an Illumina A1 sequencing adaptor (orange).

Although Mu-seq uses high-throughput NexGen sequencing, the key to capacity of Mu-seq lies in the enhanced information-return from each sequence read. This return is maximized by anchoring all Mu-seq reads to identical positions in each transposon (their TIRs), thus increasing their efficiency compared to shot-gun and/or sequence-capture approaches (See Figure 1). The resulting capacity for scale allows Mu-seq to routinely profile and map transposons in thousands of individuals simultaneously.

To enrich for sequences flanking novel insertion sites in the genome, Mu-seq employs two, nested PCR reactions to target the subset of Mutator transposons most active in maize plants that carry an autonomous MuDR transposon [11], [12]. Variation in the TIR sequences has been used to classify transposons in the Mutator (Mu) family of maize [12], [19]–[21]. We initially used conventional and 454-based sequencing of MuTAIL PCR amplicons to identify novel Mu insertion sites in the UniformMu maize population [11]–[13]. These data revealed seven TIR variants (associated with four canonical elements; Mu1, Mu7, Mu3, and Mu8) that accounted for 95% of the new Mu-transposon insertions (i.e. insertions that are unique to individual lines) captured by this approach in the UniformMu population (Figure S1). We then designed the 23 base, 12-fold-degenerate Mu-seq TIR primer to specifically target this active subset of TIR sequences.

### Construction of Multiplex Mu-seq Libraries

The protocol for construction of multiplex Mu-seq sequencing libraries is summarized in Figure 1. To obtain unbiased coverage of the maize genome, we first generate Mu-specific amplicons by ligation-mediated PCR of randomly-sheared genomic DNA. The initial round of ligation-mediated PCR is done using TIR6, a highly efficient, Mu-TIR primer that has broad specificity for Mu-family transposons [12]. A second PCR reaction uses a nested, Mu-seq primer designed to target a restricted subset of TIR sequences associated with the most active group of Mu transposons. In addition to conferring specificity, the nested Mu-seq primer incorporates a 4-base barcode and a segment of the Illumina sequencing adaptor. The 4-base, error-detecting barcode enables multiplexing of up to 64 samples in a single library. The fourth base of each code is a check-sum calculated as the sum of first three bases (where A = 0, C = 1, G = 2, T = 3) modulo 4. The check-sum enables detection of any single base sequencing error in the 4-base code. A third, 10-cycle PCR reaction is performed to complete the incorporation of the Illumina sequencing adaptor at the 5′-end of each amplicon (Fig. 1).

**Figure 2:**
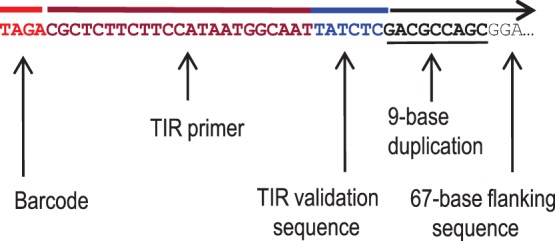
Structure of transposon-anchored Mu-seq reads. Each outward-directed, 100-base, Mu-seq sequence read includes a 27-base sequence derived from the Mu-seq adaptor [a 4-base, error-detecting, multiplex key (red), plus a 23-base, TIR-specific primer sequence (brown)]. The adjacent genomic sequence begins with 6 bases (TATCTC) from the Mu-TIR, 3′-end (blue) that are used to validate authentic Mu-flanking sequences (black). Validated reads are trimmed to remove the key and TIR sequences, then truncated if sequence quality at any point drops below a defined threshold. The remaining Mu-seq read (horizontal black arrow above sequence) thus includes one copy of the characteristic, 9-base, direct duplication created by the Mu transposon (underlined), and up to 67 bases of genomic sequence flanking the transposon insertion site (only 3 bases are shown). The barcode and library identifier are appended to the name of the trimmed read.

The structure of raw sequence reads generated by this method is shown in Figure 2. Each sequence is precision-anchored, outward oriented, and includes the 4-base barcode, plus the 23-base, TIR-specific primer sequence. The first 6 bases of genomic sequence downstream of the Mu-seq primer are derived from the highly-conserved 3′-end of the Mu-TIR. Presence of the conserved TATCTC motif at this position in the read allows validation of authentic Mu-flanking sequences by enabling detection and rejection of mis-primed sequences. When filtered on this basis, typically less than 2% of raw Mu-seq reads are rejected for mis-priming (data not shown). Additionally, up to 5% of raw reads are rejected for having unreadable or invalid barcode sequences. Validated fastq reads are trimmed to remove the barcode, adaptor and TIR sequences. Sequences with a trimmed length greater than 50 bases are converted to fasta format with the barcode sequence and library name appended to the read name.

The enhanced information content of Mu-seq reads allows extensive multiplexing of Mu-seq libraries. For our reverse genetics application in maize, we customarily use a single Mu-seq library comprised of 48 multiplexed samples to analyze 576 families (including up to 2,304 individuals). The families are grown in a 24×24 grid where each family is represented by 2 to 4 seedlings. Each of the 48 multiplexed DNA samples is obtained by pooling along one of the 24 rows or columns of the 2-dimensional grid. To ensure that only insertions of germinal origin will be represented at points of axis intersection in the grid, tissue samples for row- and column-pools are obtained from independent somatic cell lineages by taking leaves from opposite sides of each plant. In this way, non-heritable, somatic insertions will appear only in a single axis. Typically, each pool contained DNA from up to 96 seedlings. A single, multiplexed, Mu-seq library can be sequenced in one lane of a HiSeq II (Illumina) with a yield of 90 to 100 million, unidirectional, 100-base, quality-trimmed reads (about 2,000,000 reads per pool). As shown below, the resulting coverage is sufficient to detect and assign transposons to individual families. Higher dimensional designs (e.g. 4-D) using pools of up to 250 plants have also been tested successfully using this method (data not shown).

The alignment statistics for a typical MuSeq library are summarized in Table 1. About 97% of the trimmed sequences aligned to the B73 reference genome using a BLASTN cutoff score of 10^−9^. Additional filtering removed the 0.7% of alignments that had more than 4 equivalent matches in the genome and the 0.5% of alignments that did not include the 5′-end of the read, thus preventing precise mapping of the Mu insertion site. Overall, 96% of the total MuSeq reads yielded precise alignments to low-copy sequences in the B73 reference genome. Hence, sequence polymorphisms between UniformMu (W22 inbred) and the B73 inbred reference genome had little impact on alignment of MuSeq sequences.

**Table 1:** Mu-seq library alignment statistics.

	Reads	% of total reads
Total trimmed reads^1^	90,884,507	
aligned reads^1^	88,364,143	97.2
accepted alignments	87,269,249	96.0
rejected alignments: >4 copies^2^	662,509	0.7
rejected alignments: start site^3^	432,385	0.5

1 Mu-seq reads were trimmed and aligned to the maize genome as described in Materials and Methods.

2 Alignments that matched more than four sites in the genome with equivalent scores were excluded.

3 Alignments that did not begin within 10 bases of the 5-prime end of the read were excluded.

Due to the prevalence of sequence duplications in the maize genome, insertions identified by Mu-seq data do not always map to a single locus. To improve resolution of insertions that map to 2 or more locations in the genome, a “majority rule” filter is employed. If alignments identifying an insertion include more than one possible location in the genome, the site(s) supported by the greatest number of reads is chosen. Table 2 shows the effect of this filter on the distribution of insertion site copy number.

**Table 2:** Insertions with multiple map locations.

Map locations	Insertions (% of total)^1^	
	Unfiltered	Majority rule filter^2^
1	89.60	97.00
2	7.02	2.67
3	1.95	0.27
4	1.43	0.06

1 Percent of 4,723 novel insertions in the dataset shown in Table 1.

2 Majority rule filter: possible map locations are ranked by the number of reads that align at that each location. The location(s) with the highest number of reads is (are) accepted.

### Classification and Profiling of Germinal and Somatic Insertions by Mu-seq

Table 3 summarizes the profile of Mu insertion sites identified in the Mu-seq dataset presented in Table 1. The insertions fall into two broad categories depending on whether the insertion is detected in both axes of the grid or only one axis. The two-axis insertions are classified as germinal (heritable) because reads from these insertions are recovered in DNA samples from opposite leaves of each plant that derive from independent somatic lineages. In contrast, insertions detected in only one axis are classified as probable somatic insertions.

**Table 3:** Profile of Mu insertion sites detected by MuSeq.

	Insertion sites	Reads	% of total reads
Mu insertions common to all samples^1^	76	63,723,660	73.02
Mu insertions present in multiple maize lines^2^	1,808	15,290,746	17.52
Mu insertions unique to single maize lines^3^	4,723	7,906,696	9.06
Mu insertions detected in only one axis^4^	13,218	313,692	0.36
Mu insertions with <5 reads^4^	18,028	34,455	0.04
	**Total reads**	87,269,249	

1 Insertions that have reads in all 48 grid samples include endogenous Mu elements in the W22 inbred genome that are common to all plants in the population as well as insertions inherited from founder lines that are distributed widely enough within the UniformMu population to be represented in every row and column in the grid.

2 Germinal insertions that are shared by two or more maize lines in the grid are detected in multlple samples, including both axes, but do not appear in all samples. Because the number of possible locations in the grid that are consistent with multiple pairs of axis coordinates is in general larger than the number of actual locations of the insertion, these insertions cannot be unambiguously assigned to maize lines using 2D Mu-seq data alone.

3 Germinal insertions that are assigned unique locations in the grid are detected in both axes with reads occurring in a single sample of each axis.

4 Insertions due to probable somatic transpositions produce reads in only one axis. For this analysis, insertion sites within this group that were detected by fewer than 5 reads (predominantly singletons) are classified separately to distinguish them from insertion sites that have robust read counts in a single axis.

The germinal insertions fall into three classes. 1) A set of 76 insertions were present in all 48 samples indicating that they shared by most, if not all the plants in the grid. These include Mu elements that are endogenous to the W22 inbred genome and therefore shared by all plants in the population, as well as insertions inherited from earlier generations that are widely distributed in the UniformMu population [11]. 2) A second set of 1,808 germinal insertions occur at 2 or more locations within the grid, but are not ubiquitous. These include insertions inherited by plants with closely related pedigrees. Because these insertions are typically detected in both axes by more than one sample, their locations in the grid usually cannot be determined unambiguously using 2D axis intersections alone. For example, an insertion located at two positions in the grid will typically occur in two samples in each axis giving four possible pairs of axis coordinates for two actual locations. (This ambiguity is absent if the multiple lines in the grid that share an insertion happen to lie in a single row or column, but those cases are not separated out in Table 3.) 3) There were 4,723 insertions that occurred in a single sample from each axis enabling assignment to individual maize lines (see below). The distribution of MuSeq reads among these three classes of germinal insertions is in rough proportion to their relative abundance in the input DNA. For example, the concentration of Mu insertion sites that are present in every plant is 576-fold higher than the concentration of a germinal insertion site that is localized to an individual line. Insertions that are present in every plant yield an average of 838,000 total reads per insertion in the library (63,723,660 reads/76 insertions = 838,469 reads per insertion) or 1,455 reads per insertion per line (838,469 reads per insertion/576 lines = 1,455 reads/insertion/line). This is comparable to 1,674 reads per insertion per line average observed for insertions that localize to individual lines. Hence, due to their much greater abundance in the source DNA, the ubiquitous insertion sites absorb 73% of the total reads in the library.

By contrast, the set of insertions detected in only a single axis - and thus classified as putative somatic insertions - were much more numerous, but collectively accounted for less than 0.4% of the total reads (Table 3). To facilitate analysis of this group, we separated the putative somatic sites into two groups: a set of 13,218 single-axis insertions that have good sequence support (5 or more reads), and 18,218 insertion sites represented by fewer than 5 reads per insertion (predominantly single reads). Although the maize lines included in the grid were carefully screened for absence of Mu activity using the *bz1-mum9* marker, residual somatic activity can arise in at least two ways. In rare cases, Mu-active lines may escape detection if the *bz1-mum9* marker mutates to a stable loss-of-function state that is no longer responsive to MuDR. For example, Das and Martienssen [22] reported that when MuDR was present, a mutable, Mu-insertion allele of the maize *hcf106* gene produced stable, loss-of-function derivatives at a frequency of about 10^−3^ per gamete. Alternatively, we have observed that some lines that apparently lost activity through epigenetic silencing of MuDR [11] re-activate spontaneously in later generations (data not shown).

**Figure 3:**
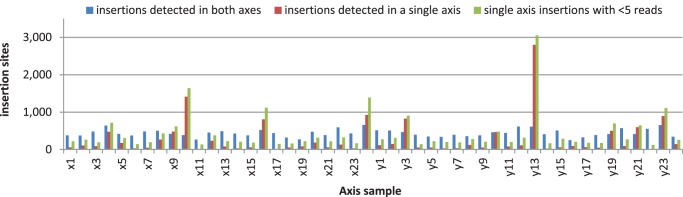
Distribution of germinal and somatic Mu insertions in Mu-seq grid samples. The bar graph shows the numbers germinal and putative somatic insertion sites detected in each of the 48 grid samples. The class of uniquely assigned germinal insertions and two classes of putative somatic insertions are described in Table 3. Whereas, the germinal insertions (blue bars) are distributed uniformly among the 48 samples, insertions detected in a single axis (red bars) and insertions with low read counts (green bars) are located predominantly in a few samples. The data are consistent with presence of at least 3 Mu-active lines in the grid indicating that our genetic screen for loss of Mu activity based on the *bz1-mum9* marker fails at a frequency of about 1% (see text). For clarity, labels of the even numbered axis samples are not shown.

To address the origin of the single-axis insertions, we examined the distribution of germinal and non-germinal insertions among the 48 DNA samples in the grid (Fig. 3). As expected, the germinal insertions were uniformly distributed over the 48 samples. By contrast, both classes of single-axis insertions were concentrated in several samples in each axis suggesting that somatic insertions generated by Mu active lines at as few as 3 locations in the grid can account for most of the single-axis insertions. The distribution of single-axis insertions with good sequence support was strongly correlated with the frequency of single-read sites indicating that both classes are most likely due to somatic insertions. These results indicate that the screen for Mu-activity based on *bz1-mum9* marker is about 99% effective. Taking into account the positive selection for loss-of-function *bz1* alleles imposed in our protocol, the 1% (∼3 of 576 lines) failure rate is compatible with the stable mutation frequency reported for the *hcf106* locus [22].

### Reproducible Detection of Insertion Sites in Complex Samples by Mu-seq

As highlighted in the previous section, reproducibility of Mu-seq is crucial for reverse genetics applications that entail mapping and profiling insertions in large collections of plants arrayed in 2D grids. Insertions must be identifiable in samples from independent axes of a grid in order 1) to identify insertions that are germinal in origin and therefore heritable, and 2) to assign the new, germinal insertions to individual maize families in the grid.

**Figure 4:**
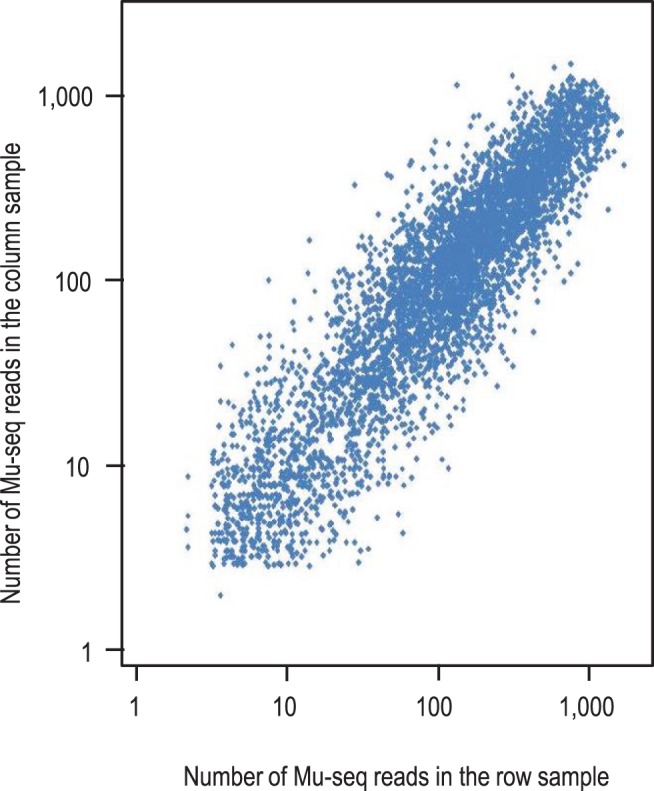
Numbers of Mu-seq reads are reproducible in DNA from independent, pooled, tissue samples. Novel transposon insertion sites were identified in a set of 576, Mu-inactive, maize families selected from the UniformMu population. The maize families were analyzed using a 48-sample, multiplex Mu-seq library sequenced in a single Illumina HiSeq II lane (see Table 1). Families represented by 2 to 4 plants were grown in a 24×24 grid array and DNA was prepared from pooled leaf samples of each row and column (24 families representing up to 96 plants per pool). Normalized numbers of Mu-seq reads are shown for row- and column- pools that yielded a total of 4,723 unique insertion sites. In 75% of instances where insertions were detected at points of axis intersection, Mu-seq read numbers differed by 2-fold or less. R^2^ = 0.72.

**Figure 5:**
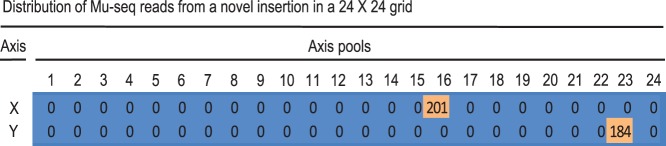
Definitive Mu-seq detection in 2-D grid axes assigns a germinal insertion to a single family. Numbers of Mu-seq reads (normalized) are shown for a typical insertion site identified in the Mu-seq, multiplex library described in Table 1. Comparable numbers of Mu-seq reads were recovered from both axes of the 24×24, 2-D grid used to sample and simultaneously analyze DNA from 576 maize families. Unambiguous localization of the example insertion (mu1050013) in independent row and column samples determined 1) that this transposon insertion was unique to a single maize family planted in position X16, Y23 of the 2-D grid, and 2) that it was germinal (heritable) because X and Y axes were sampled from opposite sides of plants (germinal, but not somatic insertions would be present in both axes). See text for further detail.

Fig. 4 shows the reproducibility of normalized read counts in X- and Y-axes for 4,723 unique, germinal insertions identified in a single 48-sample multiplex library (Table 3). To adjust for variation in sequence yields from the 48 sub-samples, numbers of these reads were normalized by the fraction of total reads that each sample contributed to the total Mu-seq library (normalized count = raw count/(sample total count/library total count/48). This normalization resulted in comparable numbers of Mu-seq reads where insertions appeared in intersecting axes of a grid. Read numbers in the intersecting axes were within 2-fold of one another for 75% of the insertions. By obtaining statistically-useful read counts with low background in both axes, Mu-seq enabled thousands of insertions to be unambiguously assigned to individual maize families (lines). Fig. 5 shows the distribution of reads for one representative germinal insertion in the 48 samples, and illustrates how this information is used to assign unique (newly created) insertions to an individual maize family. In order to select the set of 4,723 new insertions that have unique locations in the grid, the mapped insertion sites were filtered to identify those represented at a single axis intersection. As noted above, insertions present in two or more samples of both axes could not be unambiguously assigned to a unique location in the grid using a 2D design, so these were excluded from this set (Table 3).

### Base-pair Resolution of Mapped Insertions in Complex Mu-seq Datasets

**Figure 6:**
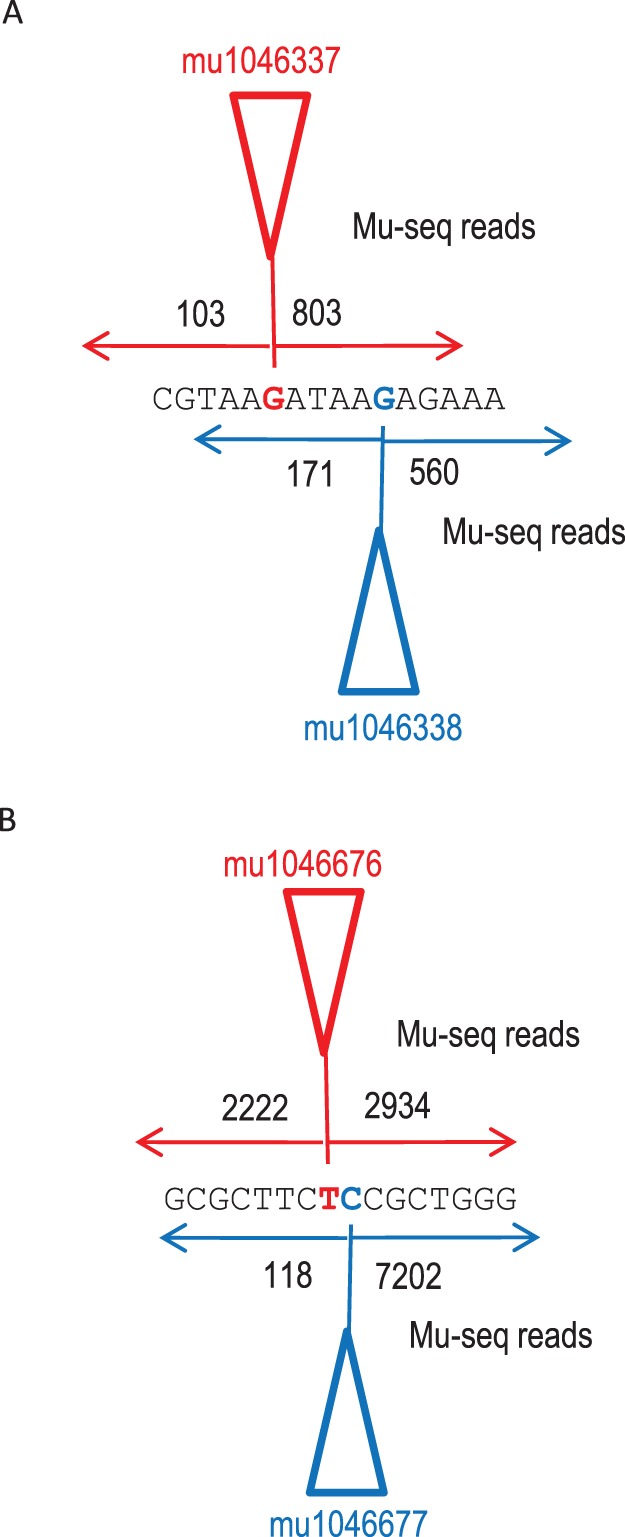
Resolution of Mu insertions from different individuals where Mu targeted nearby positions in the genome. The two pairs of insertions shown in A and B were identified in a collection of 576 families analyzed in a single Mu-seq library. A ) A pair of insertion sites separated by 5 base-pairs on Chromosome 1. Sequence is shown between positions 19,615,632 and 19,615,648. Triangles depict insertion sites (red for insertion mu1046337) (blue for insertion mu1046338). B) A pair of insertions separated by a single base-pair on chromosome 1. Sequence is shown between positions 43,421,572 and 43,421,588. Triangles depict insertion sites (red for insertion mu1046676) (blue for insertion mu1046677). Numbers adjacent to each insertion site show the quantity of Mu-seq reads recovered from forward and reverse orientations. By convention, positions of Mu insertion sites are assigned to the left-most base of the 9-base, direct duplication, and in the orientation of the maize reference genome. Accordingly, start sites for alignments of sequence reads in the reverse orientation are shifted 8-bases to the left.

By precise anchoring of each Mu-seq read to the transposon TIR, Mu-seq maximizes resolution of mapping and identification of mutations in complex samples. As shown in Figs. 6 A and 6B, the base-pair precision of Mu-seq, sequencing reads allows closely-spaced insertion sites to be distinguished from one another in the same dataset. In practice, resolution can be affected by occasional errors at the 5′-end of Mu-seq reads if these shift start points of read alignments. While the frequency of such shifts was typically less than 1 per 1,000 sequences, this reached as high as 10% for some insertion sites. The grid design allowed us to ascertain whether putative, nearby insertions sites were in fact due to distinct transposon insertions by determining whether the reads aligning to each site were derived from different locations in the grid. This appraisal indicated whether insertions occurred independently in different individuals. Two examples are shown in Fig. 6. In total, this Mu-seq dataset included 12 confirmed pairs of insertions that were separated by 2 bp, and 2 confirmed pairs separated by a single base pair.

### Sources of Variation in Read Counts among Insertions

**Figure 7:**
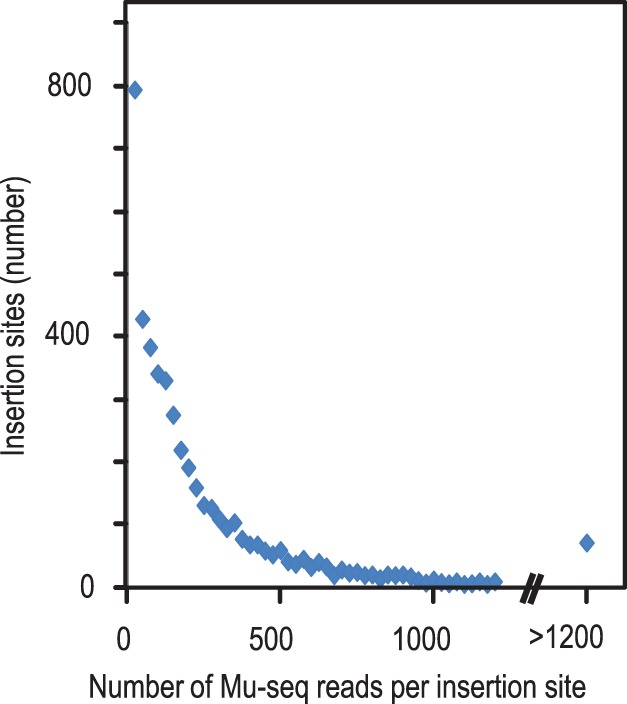
Numbers of Mu-seq reads per insertion site detected in a multiplex library. The number of reads per insertion site in the Mu-seq dataset described in Table 1 varied over 877-fold and showed an approximately exponential distribution. Because a portion of the insertions would be segregating in the four plants sampled for each family, variation in copy number can account for up to 8-fold difference; i.e. 8 copies if all four plants are homozygous for the insertion compared to 1 copy if the insertion is present in a single heterozygote. The remaining ∼110-fold range in read counts is attributed to differences in PCR amplification efficiencies of TIR-variants and flanking genome sequences (see text).

Whereas the number of sequence reads from a given insertion showed good reproducibility in independent samples, there was typically wide variation in read numbers from different insertions in the same Mu-seq profile. The dataset in Fig. 4, for example, shows that the number of Mu-seq reads from each of the new insertions detected at axes intersections varied over an 877-fold range. The distribution of insertions over this range was approximately exponential (Fig. 7). The wide variation in efficiency of detection can be attributed to at least three sources. First, a modest variation in copy number is expected due to genetic segregation of insertions in the sampled plants. In the experiment shown, segregation of new insertions in the four-seedling families would have introduced up to 8-fold variation in relative copy number for the insertion of interest (e.g. Insertions homozygous in all four plants of a given family would have eight copies, compared to one copy for insertions represented by a single heterozygous seedling). In the experiment shown, segregation was expected for roughly half of the new insertions. Second, there is variation in PCR amplification efficiency due to differences in TIR priming and/or composition of the flanking genomic sequence (e.g. G/C content). Third, some variation in mapping efficiency can result from sequence polymorphisms that impact BLAST alignment of Mu-seq reads to the reference genome. For example, indel polymorphisms near the insertion site can disrupt alignment of sequences. However, as noted in Table 1, less than 3% of Mu-seq reads from the W22 inbred background failed to align to the B73 reference genome. Overall, for the Mu-seq profiles examined here, differences in amplification and alignment efficiency were estimated to account for roughly 110-fold variation in numbers of Mu-seq reads per insertion site. We note that a comparatively small difference in amplification efficiency could contribute ∼100-fold variation in read counts. In a PCR reaction with 40 cycles, a sequence with a slightly-reduced relative efficiency of 0.89 will have less than 1% of the Mu-seq read numbers observed for a different a sequence having an efficiency of 1.0 (0.89^∧^40 = 0.009).

Such differences in sequencing efficiency for DNA flanking an insertion site may also account for the observation that a majority of insertion sites show a bias in the number of reads recovered from opposite ends of the transposon. Overall, 64% of the insertion sites showed a greater-than-10-fold bias toward one orientation. Also, 25% of the insertion sites had reads originating from just one end of the transposon. The directional bias may be due either to differences in composition of upstream and downstream flanking sequences, and/or slight variation in priming efficiency within the family of TIR sequences targeted by the Mu-seq adaptors (Methods). Evidence for both types of bias can be seen by comparing pairs of neighboring insertions that have nearly identical flanking sequences. For example, the two insertions depicted in Fig. 6A show similar biases (5-fold and 8-fold, respectively) toward the reverse orientation, which is consistent with less-efficient amplification of the downstream flanking sequence in both instances. By contrast, only one of the two insertions depicted in Fig. 6B shows a pronounced preference for one orientation, suggesting that the bias is due to TIR-related differences unique to that particular transposon rather than the composition of the flanking sequence.

## Discussion

Key features of Mu-seq that enable efficient creation and mining of transposon mutagenesis resources depend on maximizing the information obtained from every sequence read. This is achieved by anchoring each sequence to a specific site in the transposon TIR. The precision anchoring confers specificity, efficiency, and reproducibility to the mapping and identification of transposon insertions. These features, combined with throughput of the Illumina platform, allow Mu-seq to be scaled-up for simultaneous profiling of thousands of insertions in hundreds of families. The capacity of Mu-seq will thus increase proportionally with the steadily advancing throughput of NexGen sequencing platforms. In addition, by specifically anchoring reads to TIRs of active Mu transposons (including Mu1, Mu3, Mu7 and Mu8 in this instance, Fig. S1), Mu-seq reduces the background contributed by other, endogenous Mu-related sequences in the maize genome. Finally, we show that Mu-seq reads resolve different insertion sites that are separated by as little as a single base-pair, and can do so in large, complex samples. We have thus far used Mu-seq to map over 45,500 germinal Mu insertions in over 8,256 maize lines from the UniformMu transposon population (see MaizeGDB.org). Mu-seq can also be adapted for analysis of other transposons and insertional mutagens in diverse organisms.

### Mu-seq Compared to Other Methods

Alternative methods have been described for mapping native Mu insertions in the maize genome based on NexGen sequencing [14], [19]. Liu et al. [19] analyzed insertions in a large collection of maize plants using 454-sequencing. This early work was based on a single large pool that did not allow discrimination between somatic and germinal (heritable) insertions, or assignment of mapped insertions to individual plants. Nevertheless, this study marked an important first step, and the data from Liu et al. [19] provide useful insights into the diversity of active Mu elements and their insertion-site preferences in the maize genome. Williams-Carrier et al. [14] used a sequence-capture approach to enrich for Mu-flanking sequences in Illumina sequencing libraries. Their Mu-Illumina method was effective with small numbers of samples, especially in forward-genetic applications that contrasted paired samples of Mu insertions in bulked segregants from mutant and non-mutant plants. Because these Mu-Illumina reads are randomly-oriented and unanchored with respect to the TIR, multiple overlapping reads are typically required to precisely locate the insertion site in the genome. By contrast, a single Mu-seq read is generally sufficient to determine the accurate location of an insertion, hence maximizing efficient utilization and multiplexing capability of NexGen sequencing platforms. The enhanced precision of Mu-seq is particularly important in resolving nearby insertions that may occur in the same library. A second comparative advantage of Mu-seq is its scalability. Mu-seq enables simultaneous analysis and comparisons of Mu-seq profiles from hundreds of families in a single library. Finally, the Mu-seq adaptor system facilitates targeting of specific sub-families of the Mu system. Comparable selectivity may be difficult to achieve by hybridization based or sequence-capture methods.

### Targeting of Active Mu’s

In applications that use pooled samples from multiple individuals, the endogenous Mu insertion sites potentially generate a high background of flanking sequences that can limit the efficient detection of new insertions present in one or a few individuals in a pool. By using a nested primer in PCR II (See Fig. 1 and Method), we are able to focus sequencing to a subset of active Mu transposons that are controlled by MuDR. The Mu-seq, adaptor-sequence can be varied to target different sub-families of Mu elements, because the adaptor includes a region of TIR sequence that can differentiate multiple sub-families of Mu elements [12], [19]. The Mu-seq design used here targets the seven, TIR sequence variants that account for most of the new insertions detected in the UniformMu maize population. We have successfully tested several variations of the Mu-seq adaptor that target different families of Mu transposons (unpublished results).

### Classification and Profiling of Germinal and Somatic Transposon Insertions

Our Mu-seq analysis highlights the importance of incorporating genetic control of Mu activity into the design of the UniformMu population [11]. Our results indicate that selection based on the *bz1-mum9* marker enables suppression of somatic Mu-activity with about 99% efficiency. Consequently, the <0.5% of Mu-seq reads that are attributed to somatic events localize to a small number of positions in the grid. The low frequency of Mu-active escapes is consistent with the expected frequency of marker failure [22]. Because epigenetic silencing occurs in about a third of UniformMu lines [11], the possibility remains that cycling between epigenetically silenced and active states of MuDR also contributes to residual activity. Nevertheless, the exquisite sensitivity of Mu-seq enables detection of somatic insertion sites. While imposing a requirement for detection in both axes of the 2D grid provides an effective filter for identification of germinal insertions, the situation would most likely be vastly more complex without the use of marker selection to suppress somatic activity.

### Reproducibility and Quantification of Mu-seq Reads

We have shown how Mu-seq enables reproducible detection of insertion sites shared by intersecting DNA pools, and thus facilitates assignment of insertions to individuals in a 2-D grid array. Obtaining axis intersection is also crucial to filtering out non-heritable, somatic insertions which are difficult to completely eliminate genetically. Ideally, the number of Mu-seq reads could also be used to distinguish between homozygous mutants and heterozygous individuals, since these would have a two-fold difference in endogenous copies of the insertion. Although copy-number discrimination was not a goal of the experiments described here, observations indicated that routine determination of 2-fold differences would likely require a greater number of sample replicates and smaller pool sizes. Nevertheless, most mutants identified in the Mu-seq grids were represented by approximately similar numbers of Mu-seq reads in each axis (75% differing by less than 2-fold). Differences of up to 10-fold also occurred (as noted in Fig. 4). The variation may be due in part to limited replication of the large-pool samples used for grid construction, i.e. the Mu-seq profile of each axis pool is unique. However, prospects may be more promising for comparisons among replicated samples. We note that the co-efficient of variation (C.V. = standard deviation/mean) determined for Mu-seq read counts from the Mu insertion in the *bz1-mum9* mutation is 0.21 (data not shown). Because *bz1-mum9* is homozygous in all plants sequenced in our study, it is present in constant relative copy number in all of the DNA samples analyzed. The relatively low C.V. suggests that with careful sampling and replication, Mu-seq detection of a 2-fold difference in copy number per sample should be feasible.

### Opportunities for Refinement and Optimization

While Mu-seq data show good reproducibility, there is substantial variation in the number of sequence reads recovered from independent insertions. After genetic differences and sampling effects are into taken into account, estimates indicate that PCR-based biases in amplification may result in as much as 110-fold variation in numbers of Mu-seq reads. The low end of the resulting range of coverage determines the sensitivity of the method for detection of new insertions in pools of individuals. This range can be accounted for by relatively small differences in amplification efficiencies in a total of over 40 PCR cycles used in the current protocol. Refinements that reduce the number of PCR cycles may thus increase the sensitivity of Mu-seq still further for detecting rare insertions in complex DNA samples. The final output of template DNA generated by Mu-seq exceeds the requirements of the current Illumina platform, indicating room for optimization. In addition, the scalability of Mu-seq can be extended to accommodate larger as well as smaller experiments. Where greater throughput is useful, the efficiency of anchored, Mu-seq reads allows still more effective use of the Hi Seq II platform. Options include larger grids accommodated by expanding the barcodes and higher dimension grids that reduce the cost of library construction by requiring a smaller number of axis pools. We have, for example, successfully implemented 4D grids using Mu-seq (unpublished results). Alternatively, the current Mu-seq protocol is also ideal for smaller-scale experiments, and compatible with the MiSeq platform for small populations of individuals.

Finally, because the UniformMu lines used in our reverse genetics application are closely related, a large proportion of the Mu-seq reads (73%) are absorbed by Mu insertions that are common to most or all individuals in the population. Hence, a several-fold improvement in efficiency might be obtained by subtracting this comparatively small set of shared sequences from the Mu-seq library prior to sequencing. This potential gain is balanced by several considerations: 1) the added cost and complexity of library construction; 2) the possibility that subtraction (e. g. by hybridization) of shared sequences may introduce unwanted variation by suppressing other sequences in the library; 3) the shared insertions will vary in different populations, thus limiting the versatility of the method. For our current applications, these factors out-weigh the potential savings in cost of sequencing.

## Materials and Methods

### DNA Extraction and Preparation

High-quality, genomic DNA was extracted from leaf tissue that was frozen in liquid N and ground to a fine powder with a mortar and pestle as described in Suzuki et al. [23]. Depending on the experimental design, leaf samples may be prepared from an individual or a pool of 125 or more plants. DNA samples were treated with RNaseA to remove residual RNA by addition of 4 µl RNAse (1 mg/ml) to 200 µg DNA, followed by incubation for 20 min at 37°C and concentration by ethanol precipitation with ammonium acetate and resuspension in TE (pH 8.0) to a final concentration of 1 µg/µl.

For each sample, 40 µg of DNA suspended in a total volume of 200 µl was placed in a 1.5 ml TPX tube (Diagenode.com) and sonicated for two, on/off cycles of 30 s/30 s in a Biorupter UCD-200 instrument (Diagenode.com). The extent of shearing was assayed by agarose gel electrophoresis of 800 ng DNA. Fragment size was targeted for a a mid-point of about 1 kb. Because the Mu-TIR sequence may occur at any point within the randomly sheared genomic DNA fragments, the size range of Mu-anchored amplicons is expected to average about 500 bp, or half of the average size of the sheared DNA fragments. Sonication and electrophoretic analysis was repeated as needed for each sample to achieve the target size distribution. Sheared DNA samples were then concentrated by ethanol precipitation (20 µl of 3 M Na acetate, 400 µl ethanol) and re-suspended by incubation overnight in 50 µl TE. Small DNA fragments were removed by size selection with Agencourt Ampure XP Magnetic Beads (Beckman, Coulter, Inc., Brea, CA) to achieve a size cut-off of 400 bp using modifications described in the Roche, Inc. 454 FLX Titanium General Library Preparation Manual.

For subsequent DNA processing and library preparation steps, DNA samples were quantified by measuring absorbance at 260 nm with a BioRad SmartSpec 3000 or NanoDrop 1000 instrument depending on the sensitivity required.

### PCR I: Transposon-Specific, Ligation-Mediated PCR

For each sample, 3 to 5 µg of size-selected DNA fragments were made blunt ended and polished by treatment with T4 DNA polymerase and T4 polynucleotide kinase according to the manufacturer’s protocol (NEB Quick Blunting Kit). This DNA was purified using the standard protocol for the Zymo Research Zymoclean-5 DNA Clean and Concentrator Kit (ZymoResearch.com), with the following modifications: five volumes of Binding Buffer were added to each sample and DNA was eluted twice with 8 µl Elution Buffer (Zymo Research, Inc., Irvine, CA).

To prepare the double stranded adaptor for ligation to blunted ended genomic DNA fragments, the complementary strands of the adaptor oligos; tiB, 5′- CCTATCCCCTGTGTGCCTTGGCAGTCTCAG-3′ (Roche Inc., GS FLX Titanium General Library Preparation Manual) and complementary rc-tiB, 5′-CTGAGACTGCCAAGGCACAC-3′, were annealed by combining 300 µmols of each oligo (about 0.3 µl) and adding 5.4 µl of 50 mM NaCl. The oligo mixture was heated for 2 min at 95 C, and then incubated at room temperature (23°C) for 1 h. Annealed adapters were stored at −20°C.

Ligations were done using Roche Rapid DNA Ligation Kit (Roche Applied Science, Indianapolis, IN) with 200 µmol of annealed adapter and 1 µg polished DNA in a total volume of 10 µl following the manufacturer’s instructions. The reaction was incubated for 30 min at room temperature. The reaction mix was purified using the Zymo Research Zymoclean-5 DNA Clean and Concentrator Kit, as described above.

Mu flanking sequences were amplified from 200 ng of adapter-ligated DNA using a two-step, touch-down, PCR protocol with a Mu-TIR (terminal inverted repeat) specific primer TIR6, 5′- AGAGAAGCCAACGCCAWCGCCTCYATTTCGTC-3′ [12] and the adapter specific primer, tiB (see above). Amplification conditions were as follows: 1 cycle of 96°C for 3 min, followed by 10 high-stringency cycles of 96°C for 15 s, 60°C for 30 s, 72°C for 60 s, then 10 low-stringency cycles of 96°C for 15 s, 54°C for 30 s, 72°C for 60 s, and 1 cycle of 72°C for 5 min, then held at 4°C.

### PCR II: Incorporation of Nested, Multiplex, Mu-seq Adaptors

To incorporate custom, Mu-specific, multiplexed Illumina sequencing adaptors, 100 ng of PCR I amplicons were subjected to a second round of PCR using a nested Mu-seq primer that includes the 3′-end of the Illumina sequencing adaptor and an Illumina A2-tiB adaptor primer. A series of 64, Mu-seq adaptor primers were synthesized that each incorporate a 4-base barcode between a 20-base segment of the Illumina sequencing adaptor and a nested Mu-TIR primer sequence as follows: MuSeq-NNNN : 5′-ACACGACGCTCTTCCGATCTNNNNCBCTCTTCKTCYATAATGGCAAT-3′, where the barcode is indicated by N’s and the Mu-TIR specific sequence is underlined. Multiplex adaptor oligos were synthesized and HPLC-purified by MWG Operon, Inc. (Huntsville, AL) using a modified protocol to minimize cross-contamination. This series can be used to multiplex 64 samples for sequencing in a single Mu-seq and alsoenables error-detection in the multiplex code. The Illumina, A2 adaptor is incorporated at the downstream end using an A2-TiB adaptor primer: 5′-CAAGCAGAAGACGGCATACGAGATCGCCTTGGCAGTCTCAG-3′, where the TiB adaptor sequence is underlined. Amplification conditions were as follows: 1 cycle of 96°C for 3 min, followed by 10 cycles of 96°C for 15 s, 60°C for 30 s, 72°C for 90 s, then 10 cycles of 96°C for 15 s, 54°C for 30 s, 72°C for 90 s, and last, 1 cycle of 72°C for 5 min before holding at 4°C.

PCR II amplicons were purified and size-selected in a range of 250–700 bp by agarose gel electrophoresis using 1.2% agarose in TAE buffer (40 mM Tris-acetate, 1 mM EDTA). Each sample is resolved in a separate, freshly–cleaned, gel electrophoresis unit to prevent cross-contamination. The standard manufacturer protocol was used for gel purification (Zymo Research Zymoclean Gel DNA Recovery Kit), using 5 volumes of ADB binding buffer and eluting with 15 µl of DNA Elution Buffer (Zymo Research, Inc., Irvine, CA).

### PCR III - Completion of the Illumina Sequencing Adaptor System

A third PCR reaction is used to complete incorporation of the long, Illumina A1 sequencing adaptor. For each sample, a 10-cycle PCR reaction was done on 100 ng of gel-purified, PCR II amplicons using Illumina sequencing adapter primers; SolfcA_Seq1.2 5′-AATGATACGGCGACCACCGAGATCTACACTCTTTCCCTACACGACGCTCTTCCGA-3′ and f SolfcB 5′-CAAGCAGAAGACGGCATACGAGATC-3′, where the bases that overlap with PCR II primers are underlined. Amplification conditions were as follows: 1 cycle of 96°C for 3 min, followed by10 cycles of 96°C for 10 s, 61.3°C for 30 s, 72°C for 60 s, and lastly, 1 cycle of 72°C for 5 min before holding at 4°C.

The resulting template molecules were purified as per standard protocols for the Zymo Research Zymoclean-5 DNA Clean and Concentrator Kit, with the following modifications: 5 volumes of Binding Buffer were added to each sample, columns were washed twice, and eluted with 20 µl DNA Elution Buffer (Zymo Research, Inc., Irvine, CA).

To construct a Mu-seq sequencing library, 200 ng of each multiplex, template-DNA sample were pooled, and the combined volume concentrated using a Zymo Research Zymoclean-25 DNA Clean and Concentrator column (add company name, city, state). To maximize recovery and achieve a desired final concentration of at least 10 nM DNA, the columns were eluted in two steps, resulting in a combined volume of 60 µl. The first flow-through volume of the column was bound to a second column and eluted with 30 µl DNA Elution Buffer (Zymo Research, Inc., Irvine, CA), then combined with the 30 µl eluate from the first column. The resulting Mu-seq library was quantified with the NanoDrop 1000 and size distribution analyzed with a LabChip 7500.

Each library was sequenced in a single lane on the Illumina HiSeq II instrument using a uni-directional, 100-base sequencing reaction. A typical yield was 100 M reads per lane or 2,000,000 quality-trimmed reads per sub-sample in the 48-fold multiplex library.

### Bioinformatic Analysis of Mu-seq Data

Raw fastq reads were trimmed to remove 3′-adaptor sequences, if present, and to truncate each read where sequence quality dropped below a defined threshold. Sequence quality was determined beginning at the 5′ end of each Mu-seq read, and using a rolling average of base-quality scores calculated using a 10-base window. Sequences were trimmed where the rolling average fell below Q10. Each read was further screened for a valid barcode and a complete TIR sequence. The barcode sequence and library name were appended to the read name, and reads were trimmed to remove the TIR. Reads with a remaining length of at least 50 bases were converted to fasta format for BLAST analysis.

**Figure 8:**
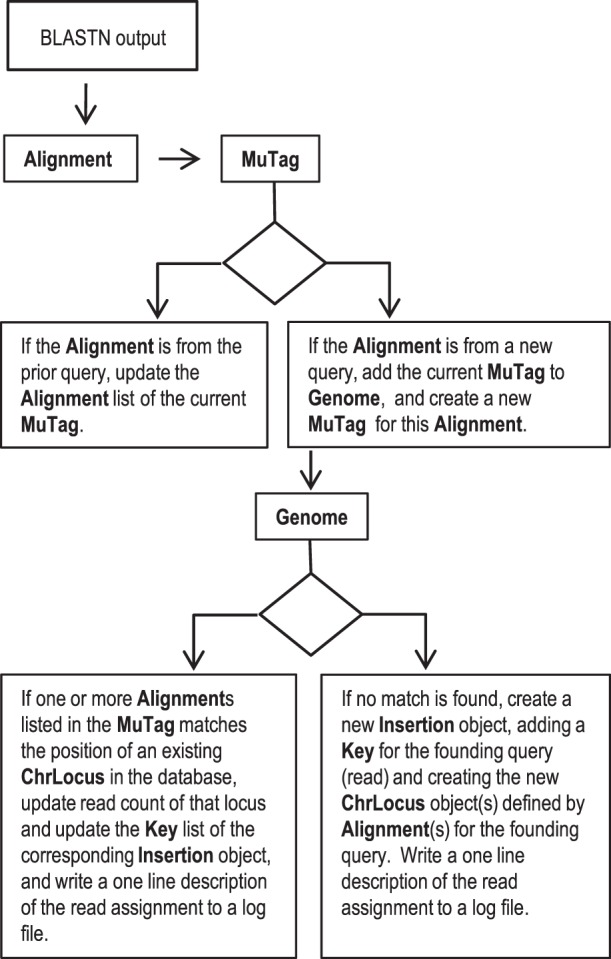
Bioinformatic analysis of Mu-seq data. Trimmed Mu-seq reads from Illumina sequencing are aligned to the B73 maize reference genome using parallel BLASTN. Tab-delimited, BLAST output is parsed into **Alignment** objects. The top scoring **Alignment**(s) for each read are stored as **MuTag** objects. The **Genome** database assigns each **MuTag** to an **Insertion** object based on matching **Alignment’**s to the chromosome location(s) (**ChrLocus** objects) associated with each **Insertion**. Allowing insertions to have multiple loci accommodates ambiguity due to duplicate sequences in the maize genome.

In order to identify and map transposon insertions, the trimmed and quality-filtered Mu-seq reads were aligned to the maize B73 reference genome by BLASTN [24]. Analyses were run in parallel processes on a 64-bit, Linux cluster (www.hpc.ufl.edu). The tab-delimited BLAST output was then parsed into a database constructed using the Java Collections framework. The software components are described in Table 4 and the work-flow is summarized in Fig. 8. Reads are mapped based on BLAST alignment(s) that have the lowest expectation score (below a cutoff of 10^−9^) and that begin within a specified distance from the 5′-end of the query sequence (the default setting is a maximum distance of 10 bp). If the alignment matches one or more of the ChrLocus positions associated with an existing Insertion Object, the read count and Key list for the matching insertion site is updated. Otherwise the alignment defines a new insertion site. By convention, insertion sites are assigned to the first base of the 9-base, direct-duplication of host-site DNA that flanks every Mu transposon in the orientation of the reference genome. To match an existing insertion, the chromosome position predicted by an alignment must be within a set distance of the consensus position of the insertion (typically this tolerance for errors at the 5′-end of the sequence is between 0 bp and 3 bp, where 0 bp requires an exact match). In addition to the chromosome location(s), each Insertion object contains a list of Key objects that record read counts for each sample that contains that insertion site.

**Table 4:** Description of Java Objects used for bioinformatics analysis of transposon insertion sites.

**Alignment**	An object describing a BLAST alignment of a sequence read to the reference genome. Each line of tab-delimited BLAST output (i.e. set by BLAST option -outfmt 6) generates an Alignment object.
**MuTag**	An object that contains a list of the best scoring Alignment’s for a sequence query. I.e. each unique query (read) in the BLAST output generates a MuTag object. Only Alignment’s that have scores equal to top scoring Alignment for the query are retained.
**Genome**	A java Collections based database for Mu insertion data that contains a set of ordered lists of ChrLocus objects, one for each chromosome in the reference genome plus unplaced sequences, and a list of unique insertions (represented by Insertion objects) that have been mapped in the genome.
**Insertion**	A class describing the chromosome location(s) of a unique, mapped insertion site and the libraries and samples that contain reads from that insertion site. Its members include a unique identifier (e.g. muXXXXXX), a list of ChrLocus objects that describe possible locations of the insertion in the genome, and a list of Key objects that identify libraries and samples that contain reads from this insertion site. The ChrLocus list accommodates the fact that due to complexity of the genome (duplicated genes, etc) and limited read length some insertion sites map to multiple unresolved locations.
**ChrLocus**	An object describing a unique location in the reference genome. Member fields describe the chromosome and base pair position of a unique locus, a total count of the reads that have been mapped to that locus, and a count of reads that align in reverse orientation (allowing calculation of forward read count by subtraction). In addition, it contains a reference to the Insertion (unique insertion site) that is associated with this locus. A ChrLocus is uniquely assigned to one Insertion.
**Key**	An object that contains a count of reads assigned to each sample in a multiplex library. Member fields include the four-base barcode sequence, a unique library name, and a count of reads in the library that have that barcode.

To accommodate reads that have multiple equivalent alignments in the genome, each Insertion object contains a list of possible locations (ChrLocus objects) for the insertion. For the reverse genetics application described here for Mu-seq, we exclude reads that align with equal scores to more than four locations in the genome. Using that cutoff, about 10% of Insertion objects in the database contain more than one possible location in the genome and less than 4% have more than two unresolved locations (Table 2). In instances where there are multiple locations, the list of locations may be ranked according to the number of reads that align at each location. If this “majority rule” criterion is applied (Table 2), then 97% of insertions can be assigned to a unique location and less than 0.5% of insertions have greater than 2 unresolved locations in the genome. All of the java objects implement the Serializable interface enabling the database to be stored as a serialized java object file.

### Access to Mu-seq Sequences

The sequences are deposited in the NCBI Sequence Read Archive as accession SRP028545.

## Supporting Information

Figure S1 **Classification of TIR sequences from Mu transposons active in the UniformMu population.** To survey active Mu elements, amplicons that included the 3′-end of the Mu-TIR, plus flanking genomic sequence were prepared from a set of 96 UniformMu families and sequenced in this initial work by 454 (Roche, Inc). The amplicons were generated by ligation-mediated PCR using nested, Mu-TIR primers that have broad specificity (TIR6 and TIR4; Settles et al., 2004). The TIR sequences for unique insertions were classified based on comparison of their 3′-most, 28 bases to those of TIR sequence variants characteristic of canonical Mu-family elements (Mu1 through Mu11 and MuDR). The “other” category includes less common TIRs as well as variants due to probable sequencing errors. TIR variants associated with the abundant Mu elements (Mu1, Mu7, Mu8, Mu1 and Mu3) were used to design the Mu-seq adaptor.
(EPS)

## References

[1] Spradling AC, Stern DM, Kiss I, Roote J, Laverty T, et al (1995) Gene disruptions using *P* transposable elements: an integral component of the *Drosophila* genome project. Proc. Natl. Acad. Sci. USA 92: 10824–10830. 10.1073/pnas.92.24.10824 PMC40524 7479892

[2] Thibault ST, Singer MA, Miyazaki1 WY, Milash B, Dompe NA, et al (2004) A complementary transposon tool kit for Drosophila melanogaster using P and piggyBac. Nature Genetics 36: 283–287. 14981521 10.1038/ng1314

[3] Alonso JM, Stepanova AN, Leisse TJ, et al.. (2003) Genome-wide insertional mutagenesis of Arabidopsis thaliana. Science, 301, 653–657. 10.1126/science.1086391 12893945

[4] O’Malley RC, Ecker JR (2010) Linking genotype to phenotype using the Arabidopsis unimutant collection. Plant J. 61: 928–40. 10.1111/j.1365-313X.2010.04119.x 20409268

[5] Miyaoa A, Tanakab K, Muratab K, Sawakib H, Takedaa S, et al (2003) Target Site Specificity of the Tos17 Retrotransposon Shows a Preference for Insertion within Genes and against Insertion in Retrotransposon-Rich Regions of the Genome. Plant Cell 15: 1771–1780. 12897251 10.1105/tpc.012559 PMC167168

[6] Hirochika H, Guiderdoni E, An G, Hsing YI, Eun MY, et al (2004) Rice mutant resources for gene discovery. Plant Mol Biol 54: 325–34. 15284490 10.1023/B:PLAN.0000036368.74758.66

[7] Qu S, Desai A, Wing R, Sundaresan V (2008) A versatile transposon-based activation tag vector system for functional genomics in cereals and other monocot plants. Plant Physiol. 146: 189–99. 10.1104/pp.107.111427 PMC2230568 17993541

[8] Bensen RJ, Johal GS, Crane VC, Tossberg JT, Schnable PS, et al (1995) Cloning and characterization of the maize *An1* gene. Plant Cell 7: 75–84. 7696880 10.1105/tpc.7.1.75 PMC160766

[9] May BP, Liu H, Vollbrecht E, Senior L, Rabinowicz PD, et al (2003) Maize-targeted mutagenesis: A knockout resource for maize. Proc Natl Acad Sci USA 100: 11541–6. 12954979 10.1073/pnas.1831119100 PMC208794

[10] Fernandes J, Dong Q, Schneider B, Morrow DJ, Nan GL, et al (2004) Genome-wide mutagenesis of Zea mays L. using RescueMu transposons. Genome Biol. 5: R82. 10.1186/gb-2004-5-10-r82 PMC545602 15461800

[11] McCarty DR, Settles AM, Suzuki M, Tan BC, S Latshaw, et al (2005) Steady-state transposon mutagenesis in inbred maize. Plant J 44: 52–61. 16167895 10.1111/j.1365-313X.2005.02509.x

[12] Settles AM, Latshaw S, McCarty DR (2004) Molecular analysis of high-copy insertion sites in maize. Nucleic Acids Res 32: e54. 15060129 10.1093/nar/gnh052 PMC390377

[13] Settles AM, Holding DR, Tan BC, Latshaw SP, Liu J, et al (2007) Sequence-indexed mutations in maize using the UniformMu transposon-tagging population. BMC Genomics 8: 116. 17490480 10.1186/1471-2164-8-116 PMC1878487

[14] Williams-Carrier R, Stiffler N, Belcher S, Kroeger T, Stern DB, et al (2010) Use of Illumina sequencing to identify transposon insertions underlying mutant phenotypes in high-copy Mutator lines of maize. Plant J. 63: 167–77. 10.1111/j.1365-313X.2010.04231.x 20409008

[15] Cresse AD, Hulbert SH, Brown WE, Lucas JR, Bennetzen JL (1995) Mu1-related transposable elements of maize preferentially insert into low copy number DNA. Genetics. 140: 315–24. 10.1093/genetics/140.1.315 PMC1206558 7635296

[16] Chandler VL, Hardeman JK (1992) The Mu elements of Zea mays. Adv Genet 3: 77–122. 10.1016/s0065-2660(08)60319-3 1333722

[17] Lisch D, Chomet P, Freeling M (1995) Genetic characterization of the Mutator system in maize: behavior and regulation of Mu transposons in a minimal line. Genetics 139: 1777–96. 7789777 10.1093/genetics/139.4.1777 PMC1206502

[18] Schnable PS, Ware D, Fulton RS, Stein JC, Wei F, et al (2009) The B73 maize genome: complexity, diversity, and dynamics. Science. 326: 1112–5. 10.1126/science.1178534 19965430

[19] Liu S, Yeh CT, Ji T, Ying K, Wu H, et al (2009) Mu transposon insertion sites and meiotic recombination events co-localize with epigenetic marks for open chromatin across the maize genome. PLoS Genet. 5: e1000733. 10.1371/journal.pgen.1000733 PMC2774946 19936291

[20] Hunter CT (2010) The Mu transposons of *Zea mays* and their use in determining gene function: Cellulose synthase-like D genes in plant and cell development. University of Florida. 141 p.

[21] Tan BC, Chen Z, Shen Y, Zhang Y, Lai J, et al (2011) Identification of an active new mutator transposable element in maize. G3 (Bethesda) 1: 293–302. 22384340 10.1534/g3.111.000398 PMC3276141

[22] Das L, Martienssen RA (1995) Site-selected transposon mutagenesis at the hcf106 locus in maize. Plant Cell. 3: 287–294. 10.1105/tpc.7.3.287 PMC160782 7734963

[23] Suzuki M, Settles AM, Tseung CW, Li QB, Latshaw S, et al (2006) The maize viviparous15 locus encodes the molybdopterin synthase small subunit. Plant J 45: 264–74. 16367969 10.1111/j.1365-313X.2005.02620.x

[24] Altschul SF, Gish W, Miller W, Myers EW, Lipman DJ (1990) Basic local alignment search tool. J Mol Biol. 215: 403–10. 10.1016/S0022-2836(05)80360-2 2231712

